# Microglial VPAC1R mediates a novel mechanism of neuroimmune-modulation of hippocampal precursor cells via IL-4 release

**DOI:** 10.1002/glia.22682

**Published:** 2014-06-18

**Authors:** Robert Nunan, Harri Sivasathiaseelan, Damla Khan, Malik Zaben, William Gray

**Affiliations:** 1 Division of Clinical Neurosciences, University of SouthamptonSouthampton, United Kingdom; 2 Institute of Psychological Medicine and Clinical Neurosciences, National Institute for Neuroscience and Mental Health Research, Cardiff UniversityCardiff, United Kingdom

**Keywords:** neurogenesis, microglia, VIP, cell culture

## Abstract

Neurogenesis, the production of new neurons from neural stem/progenitor cells (NSPCs), occurs throughout adulthood in the dentate gyrus of the hippocampus, where it supports learning and memory. The innate and adaptive immune systems are increasingly recognized as important modulators of hippocampal neurogenesis under both physiological and pathological conditions. However, the mechanisms by which the immune system regulates hippocampal neurogenesis are incompletely understood. In particular, the role of microglia, the brains resident immune cell is complex, as they have been reported to both positively and negatively regulate neurogenesis. Interestingly, neuronal activity can also regulate the function of the immune system. Here, we show that depleting microglia from hippocampal cultures reduces NSPC survival and proliferation. Furthermore, addition of purified hippocampal microglia, or their conditioned media, is trophic and proliferative to NSPCs. VIP, a neuropeptide released by dentate gyrus interneurons, enhances the proliferative and pro-neurogenic effect of microglia via the VPAC1 receptor. This VIP-induced enhancement is mediated by IL-4 release, which directly targets NSPCs. This demonstrates a potential neuro-immuno-neurogenic pathway, disruption of which may have significant implications in conditions where combined cognitive impairments, interneuron loss, and immune system activation occurs, such as temporal lobe epilepsy and Alzheimer's disease.

## Introduction

Postnatal and adult hippocampal neurogenesis support hippocampal dependent learning and memory under physiological conditions (Dupret et al., 2007; Gould et al., 1999; Leuner et al., 2006; Shors et al., 2001; Winocur et al., 2006; Zhang et al., 2008). Furthermore, altered neurogenesis has been implicated in cognitive and mood impairment in temporal lobe epilepsy (Barkas et al., 2012; Kuruba et al., 2009), depression, and certain neurodegenerative diseases (Jessberger et al., 2007; Jin et al., 2004; Kempermann, 2002; Malberg et al., 2000; Parent et al., 1997; Rockenstein et al., 2007), suggesting that hippocampal neurogenesis may be an appropriate therapeutic target for these conditions. All these conditions have an important neuroinflammatory component to their pathogenesis, and the innate and adaptive immune systems are increasingly recognized as important control systems for hippocampal neurogenesis under both physiological and pathological conditions. However, the mechanisms by which the immune system regulates hippocampal neurogenesis are incompletely understood.

It is clear that in the stem cell niche there is a dynamic interplay between neural stem/progenitor cells (NSPCs) and microglial cells. Unchallenged microglia play an important role in scavenging apoptotic precursors in the subgranular zone niche (Sierra et al., 2010), and *in vitro* studies have shown that TGFβ released by scavenging microglia promotes stem cell/neuronal proliferation and survival (Battista et al., 2006; Fadok et al., 1998). Microglia through either the release of pro-inflammatory agents or neurotrophic factors (IGF1, BDNF, etc.) can affect neurogenesis as well as neuronal survival and function (Battista et al., 2006; Butovsky et al., 2006; Cacci et al., 2008; Ekdahl et al., 2003; Ziv et al., 2006). More recently, it has become apparent that neurons themselves may control and regulate the immune activation and function, including microglia, either directly or through mediators (Biber et al., 2007; McAllister and van de Water, 2009; Pocock and Kettenmann, 2007; Zietlow et al., 1999). Within the stem cell niche, GABA-ergic interneurons are ideally placed to signal neuronal activity and to co-release important peptide neurotransmitters such as NPY and VIP into the surrounding microenvironment. Although GABA has been shown to promote neuronal differentiation of NSPCs and the integration of their progeny, we have previously shown that NPY, co-released by GABA-ergic interneurons under certain firing conditions, is a potent proliferative factor for NSPCs in the postnatal and adult dentate (Howell et al., [Bibr b34],[Bibr b33],[Bibr b35]) and that Galanin is both proliferative and trophic for neuronally committed precursors (Abbosh et al., 2011). We have also shown that VIP through VPAC2 receptor activation expands the pool of proliferating nestin-expressing dentate NSPCs, by preventing either a neuronal or glial fate choice and by separately supporting their survival, while selective VPAC1 receptor activation promotes a neurogenic granule cell fate (Zaben et al., 2009).

Although VIP as a neurotransmitter is a direct regulator of hippocampal neurogenesis (Zaben et al., 2009), it exerts a wide spectrum of immunological functions controlling the homeostasis of the immune system with a primary anti-inflammatory role (Gomariz et al., 2001). In adaptive immunity, VIP appears to inhibit cytokine production and proliferation of T-lymphocytes, as well as inducing Th2 differentiation of T-lymphocytes, rather that Th1 (Gonzalez-Rey and Delgado, 2007; Gonzalez-Rey et al., 2007). In innate immunity, VIP inhibits several macrophage functions, including phagocytosis, respiratory burst, and chemotaxis as well as LPS-induced production and release of pro-inflammatory cytokines (IL6, TNFα, and IL12) and chemokines (Delgado et al., 2003). Although it is clear that VIP reduces release of inflammatory mediators that are detrimental to neurogenesis from LPS-activated macrophages and microglia, the effect of VIP on “unstimulated” microglia has not been elucidated. The presence and release of VIP within the lymphoid microenvironment and the existence of VIP receptors on immune cells suggest that neuropeptides such as VIP are prime candidates for being a mediator of neuro-immune interactions (Ganea and Delgado, 2001). As microglia express neuropeptide receptors (Pocock and Kettenmann, 2007), it is possible that this neuro-glial interaction is mediated by neuropeptides; however, information regarding the effects of neuropeptides on microglial activation is sparse and as a result the effects of neuropeptides on microglial-derived cytokines, chemokines, and neuroprotective factors are not fully understood.

To study the role of VIP as a component of the neuro-immune modulation of hippocampal neurogenesis, we have generated postnatal hippocampal neural stem cells (when dentate gyrus neurogenesis at its peak) and either co-cultured them with pure hippocampal microglia or treated them with microglia-conditioned medium in the presence or absence of VIP. We show that depletion of microglia from cultures is associated with reduced NSPC survival and proliferation, while co-culturing of neural stem cells with microglia or treatment with microglia conditioned medium enhances NSPC survival and proliferation. We further demonstrate that treatment of NSPCs with conditioned medium of microglia pre-exposed to VIP at physiological concentrations significantly enhances the proliferative and trophic effect of microglia. These VIP-stimulated microglia-mediated proliferative effects on NSPCs are mediated via the release of the anti-inflammatory cytokine IL-4, as blockade of IL-4 completely abolished the proliferative effects of microglial VIP pre-conditioning. This study identifies the neuropeptide VIP as an important neuro-immune modulator in the control of hippocampal neurogenesis as well as emphasizing the role of microglia as an important member of the neural stem cell niche.

## Materials and Methods

### Cell Culture

Postnatal rat hippocampal NSPC cultures were generated from postnatal Wistar rat hippocampi (P7-10) as described elsewhere (Howell et al., 2003). Briefly, under sterile techniques, hippocampi were quickly dissected and sectioned on a MacIlwain tissue chopper into 400-μm-thick slices in GEY's balanced salt solution (Life Technologies, Paisley, UK) supplemented with 4.5 mg/ml glucose at 4°C. The tissue slices were then digested with 2 mg/ml papain (22.0 U/mg, Sigma) in pre-warmed Neurobasal A, supplemented with 2% B27 (Life Technologies) and 0.5 mM Glutamine (Sigma, UK) for 30 min at 37°C. After washing, cell release was achieved by trituration 10–15 times with a Pasteur pipette in NB-A/B27 Glutamine medium. Progenitor cells were purified free of debris and enriched on a two-step density OptiPrep (Axa-Shields, Oslo, Norway) gradient. Viable cells were then seeded at a density of 100,000 viable cells per ml in NB/B27 and Glutamine directly onto poly-l-lysine coated 24-well plates and cultured in a humidified incubator at 37°C (5% CO_2_/95% Air). At 2 h after plating the medium was replaced by fresh medium of NB/B27 and Glutamine or experimental condition. All media included a combined antibiotic/antimycotic (Penicillin/Streptomycin and Fungizone, Life Tech). For cultures longer than 3 days, two-third of the culture medium was replaced every 3 days.

### Generation of Pure Microglial Cultures

Mixed hippocampal cells were isolated as described above. Viable cells were plated in 1 ml/well culture medium at a density of 5 × 10^5^ cells/ml directly onto poly-l-lysine coated six-well plates (Corning). Two hours after plating, the cells were washed and replenished with fresh culture medium supplemented with 10% fetal bovine serum (FBS) and 1% antibiotic/antimycotic. After 7 days the plate was then shaken at 1,200 rpm for 10 min followed by several hard taps on the side and 5 s of swirling to dislodge weakly attaching cells, which are primarily microglia. The cell suspension from several wells was pooled and re-plated onto fresh uncoated six-well plates at a concentration of 1.5 × 10^5^ viable cells/ml in hippocampal culture media. Cells were allowed to attach for 1 h, after which they were washed with fresh medium to remove FBS and weakly attached cells. Purity was determined using FITC conjugated IB4 and phase contrast imaging.

To generate microglia conditioned medium, after the purified microglia had been incubated for 24 h in culture medium or experimental condition, the medium was collected and passed through a 0.22 μm sterile filter. Microglia conditioned medium (500 μl/well) was then added to primary hippocampal cultures at 2 h post-plating.

To generate pure microglial cell suspension for co-culture, 1 ml of warm Trypsin-EDTA was added to the freshly purified microglia for 5 min at 37°C. FBS (10%) was then added to inhibit any further Trypsin activity. Cells were dislodged of the plate by triturating the cells. Cell suspension was collected and centrifuged at 1,100 rpm for 2 min. The supernatant was removed and the cell pellet re-suspended in 3 mL of hippocampal culture medium. The process was repeated once more. The resultant cell suspension was diluted to 1 × 10^5^ cells/ml. Cell suspension (500 μl/well) was then added to primary hippocampal cultures at 2 h post-plating.

### Depletion of Microglia from Cultures

Microglia present in primary hippocampal neuronal cultures were selectively killed using rat-MAC-SAP (Advanced Targeting Systems). MAC-SAP is a microglia specific antibody (binds to CD11b) conjugated to saporin (a ribosome inactivating protein). This results in only cells expressing CD11b, namely microglia, being targeted by the toxin.

Rat-MAC-SAP (100 μg/ml), in culture medium, was added to hippocampal neuronal cultures 2 h post-plating. Hippocampal cells were then incubated for 24 h with the rat-MAC-SAP. At 1DIV, the cells were washed and fresh warmed culture medium added.

### Pharmacology

To examine VPAC1 and/or VPAC2 mediation of VIP effects in culture, we used the VPAC1 receptor selective agonist [Lys15, Arg16, Leu27]-VIP (1–7)—GRF (8–27) (30 nM, Phoenix Pharmaceutical, INC) and the VPAC1 selective antagonist [Ac-His1, D-Phe2, Lys15, Arg16, Leu27]-VIP (3–7)-GRF (8–27) (1 μM, Phoenix Pharmaceutical, INC) for the indicated times.

### IL-4 Treatment and Blockade

The role of interleukin-4 in mediating the effects of VIP-treated microglia on hippocampal progenitor cells was investigated by treating cells with 10 ng/ml purified neutralizing antibody against the alpha subunit of the IL4 receptor (IL-4Ra) [rat-anti-mouse CD124-(BD Pharmingen)] to block interleukin-4 activity.

To investigate the proliferative effect of interleukin-4 on hippocampal progenitor cells, cells were cultured for 3DIV and for the terminal 8 h prior to fixation, treated with 10 ng/ml recombinant rat IL4 (R&D Systems).

### Cell Death Quantification

Cell death in culture was quantified using the cell death marker propidium iodide (PI) and the nuclear stain 4′,6-diamidino-2-phenylindole (DAPI). PI a highly polar fluorescent dye, which is normally excluded from healthy cells, enters dead or dying cells with leaky membranes. Once inside the cells PI binds to nucleic acids in both the cytoplasm and nucleus, making the cells highly fluorescent at wavelengths of 490 nm excitation and 590 nm emission. DAPI is a nuclear cell stain that binds to double-stranded DNA by forming a stable fluorescent complex with a blue stain. However, DAPI is a non-polar dye that enters both healthy (intact cell membrane) and dying cells (damaged cell membrane). Therefore, the number of cells stained with DAPI indicates the total number of cells in culture. From methodological point of view, PI was added to living cells (not fixed) in cultures at 5 μg/ml for 40 min at 37°C. Cells were (while still alive) then incubated in NB/B27 and Glutamine containing DAPI (20 μg/ml) for another 40 min. Finally, DAPI containing medium was removed and cells were maintained in fresh culture medium (NB/B27 and Glutamine) while being imaged for quantification. The proportions of non-viable cells (PI stained) of the total (DAPI stained) cell population were then calculated.

### Assessment of Cell Proliferation

In our cell culture system, we used bromodeoxyuridine (BrdU) to measure cell proliferation. BrdU is a thymidine analog that is incorporated into the DNA of proliferating cells during the S-phase of the cell cycle in immunohistochemically detected amounts (Kuhn and Cooper-Kuhn, 2007). It has been shown that the length of the cell-cycle of progenitor cells is 12–14 h and S-phase is estimated to correspond to a third or a half of the cell cycle. Therefore, cells were cultured for 3DIV and 20 μM BrdU was added to the cells for the terminal 8 h before rinsing the cells once in PBS and then fixing them in 4% PFA for 30 min at 4°C. Immunohistochemistry could then be used to quantify BrdU incorporation.

To study the proliferation of progenitor cells (our primary cells of interest), we stained for BrdU and nestin (as a marker of putative progenitor cells). An increase in the proportion of nestin-expressing cells that incorporate BrdU (known as the mitotic index) indicates increased proliferation of neural progenitors.

Cell proliferation was further studied using polyclonal antibodies against the proliferation antigen Ki67, in combination with BrdU. Ki-67 is an endogenous marker that is expressed by proliferating cells during active phases of the cell cycle: lateG1, G2, and M phase, but not detectable in quiescent cells (G0 phase) (Kee et al., 2002). Using BrdU, Ki-67, and DAPI we measured the labeling index (cell cycle speed) and the growth fraction (recruitment of quiescent cells) of cells in culture.

### Immunohistochemistry

At the indicated time for each experiment, immunoflourescent staining was performed on paraformaldehyde (PFA)-fixed cells. For this study, the following primary antibodies were used in PBS-0.1% Triton-X: rat anti-BrdU diluted 1:200 (Insight Biotechnology), mouse anti-rat nestin diluted 1:200 (BD Biosciences), rabbit anti-GFAP diluted 1:500 (DAKO), mouse anti-TuJ1 diluted 1:500 (Sigma), rabbit anti-class III-β-tubulin diluted 1:500 (Chemicon), and/or rabbit anti-Ki67 diluted 1:500 (Novocastra). Primary antibodies were probed using Cy2 or Cy3-conjugated anti-rat diluted 1:500, anti-mouse diluted 1:500, and/or anti-rabbit diluted 1:200 secondary antibodies (Jackson ImmunoResearch). Cells were then counterstained with the nuclear stain DAPI (5 μg/ml) (Sigma). For BrdU immunostaining, DNA was first denatured by incubating the cells in 2 M HCl at 37°C for 30 min.

### Data Analysis

Imaging was performed on an inverted DM IRB microscope (Leica Microsystems UK, Milton Keynes, UK). The area of a 20× field was measured using a 255 μm grid graticule slide (Microbrightfield, Williston). Cell counting was performed on six random 20× fields per well using the Open Lab image-capturing system version 2.1 (Improvision, Lexington, MA). Raw data from the 20× field counts were averaged and plotted ± SEM and expressed as cells/mm^2^ per well, based on a sample of four to eight wells per condition per experiment. All experiments were repeated at least three times. One experiment consisted of four hippocampi from two animals, pooled and prepared as described above. Data points were plotted using GraphPad Prism data analysis software (GraphPad). The statistical significances between the means were assessed by either Student's *t* test for single comparisons or by ANOVA followed by post hoc tests for multiple comparisons, with *P* < 0.05 considered significant.

### PCR Assay

For PCR, total RNA was extracted from cultured cells and directly reverse-transcribed to complementary DNA (cDNA) using SuperScript™ III Cells Direct cDNA Synthesis Kit (Invitrogen™ Life Technologies). The cDNA was then amplified by one-step PCR kit (rat Custom real-time PCR assay for use with SYBRgreen chemistry) (PrimerDesign, Southampton) in a real-time thermocycler (Rotor-Gene 6000, Corbett Robotics). The PCR reaction amplification conditions were: enzyme activation for 10 min at 95°C followed by 15 s for denaturation at 95°C and then the data were collected in 60 s at 60°C. VIP receptor primers were: VPAC1; Forward: TAACTGAAGCGGGTGTGGAT and Reverse: CCTCTCCTAGCCCTCAAACA. VPAC2; Forward: CGGATTTCATAGATGCGTGTG, Reverse: CACTGTAGCCCAAGGTATA AAATG. For non-quantitative PCR, reaction amplifications for each product were then run on 2% agarose gel containing ethidium bromide and visualized using UV light. To further verify gene expression in these cultures, we used quantitative PCR where reactions were performed with 1 μL of the reconstituted primer mix, 10 μL of PrimerDesign 2X Precision™ MasterMix with SYBRgreen and 4 μL PCR-Grade water to which 25 ng/μL of the indicated (DG *vs*. HSVZ or control *vs*. VIP) cDNA was added to a final volume of 20 μL. Fluorescent data were collected at once during each cycle of amplification which allowed us for real time monitoring of the amplification. Data were automatically normalized and a threshold at was set at the level where the rate of amplification is the greatest during the exponential phase. Ct-values were collected, raw data were processed and analyzed using the Comparative Ct method (Schmittgen, 2001) where the comparative expression level equals to 2^−ΔΔCt^. For this method of analysis to be successful, a validation experiment is required. This validation experiment was performed to confirm that the amplicons performed equally efficiently across the range of the initial template amounts (Bustin, 2000).

## Results

### Microglia Are Present In Hippocampal Cultures and Their Depletion Increases Cell Death and Reduces NSPC Proliferation

Hippocampal progenitor cultures were generated from postnatal rats [as described by Howell et al. (2003)] and grown for 5DIV before being processed for the presence of microglia, using the microglia-specific marker IB4 (Dailey and Waite, 1999). The mean density of microglia in our cultures was 18.89/mm^2^ and accounted for 11.38% of all cells in culture (data not shown). Microglia were highly motile and exhibited a dynamic phenotype (observed by time-lapse photography, not shown). The majority of microglia were amoeboid in shape reflecting an activated phenotype supported by CD68 staining which detects lysomes.

**Figure 1:**
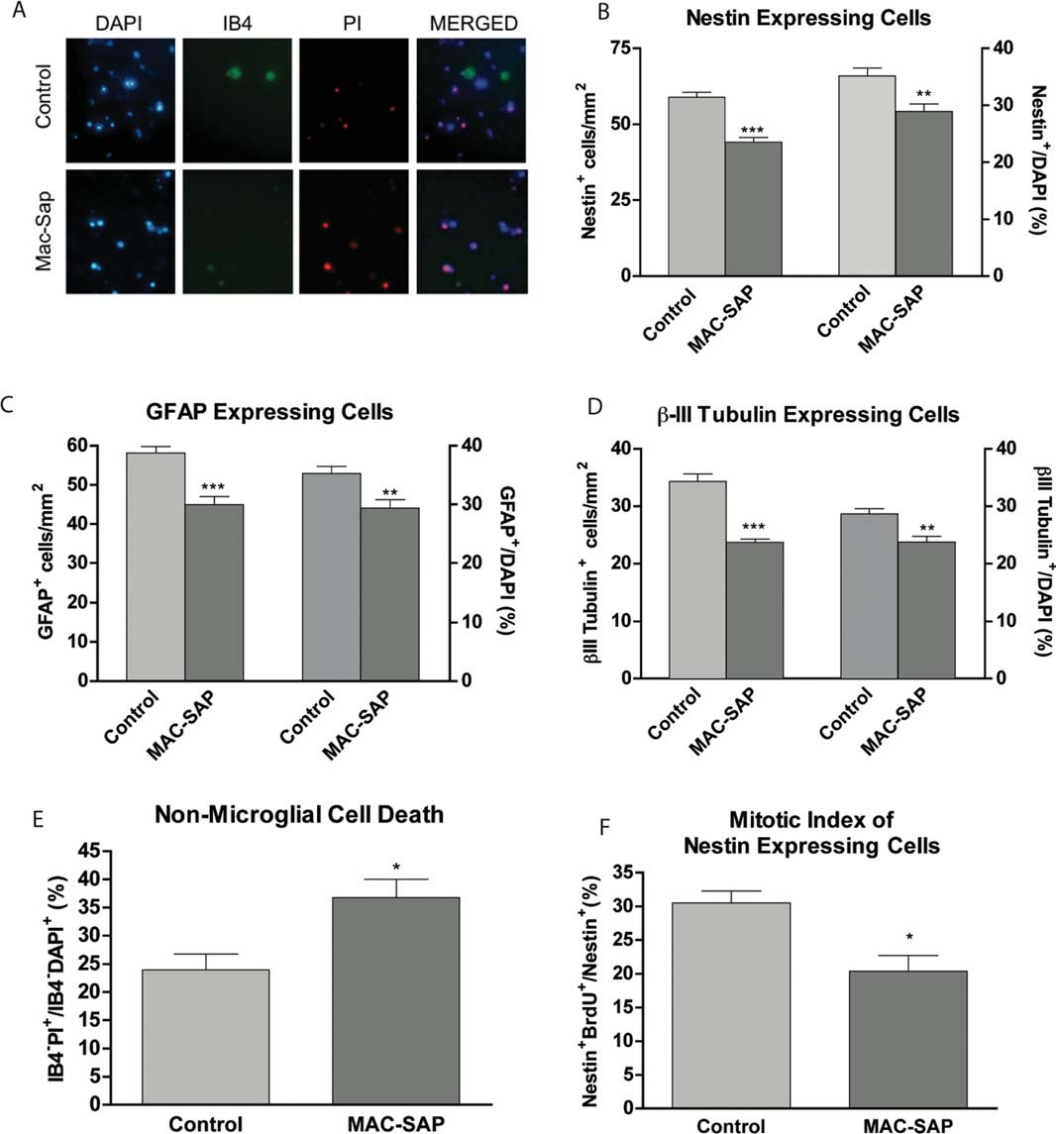
Depletion of microglia from hippocampal progenitor cell cultures results in increased cell death and reduced proliferation. Hippocampal progenitor cell cultures were generated from postnatal rats. Cells were grown for 1DIV in the presence or absence of 100 ng/ml MAC-SAP. Cells were then washed to remove MAC-SAP and fresh medium was added. A: At 5DIV dead or dying microglia were confirmed with IB4 and PI co-expression. B: At 5DIV total number and percentage of Nestin, C: GFAP, and D: Class III-β-Tubulin cells were quantified in control or MAC-SAP treated conditions. E: At 5DIV, IB4, DAPI, and PI were added for the final 3 h prior to one wash and imaging was performed. The percentage of non-microglial cells that are dead (IB4^−^PI^+^/IB4^−^DAPI^+^) was calculated. F: 20 μM BrdU was added to cells at 3DIV for 8 h. Cells were then fixed and stained for BrdU and Nestin. The mitotic index (expressed as a percentage of nestin^+^ cells that are also BrdU^+^) was calculated. *P < 0.05, **P < 0.01, ***P < 0.001 (Student's *t*-test).

We next sought to examine the role of microglia in the survival and proliferation of hippocampal progenitor cells by depleting our cultures of microglia, using rat-MAC-SAP (a microglia-specific antibody conjugated to the toxin saporin). At 5DIV MAC-SAP treatment killed 85% of endogenous microglia as demonstrated by IB4 and PI co-staining (Fig. 1A). We found that removal of microglia from cultures resulted in a decrease in both the number and percentage of nestin-expressing cells by 14.81 ± 2.25 cells/mm^2^ and 6.25 ± 1.87%, respectively (Fig. 1B). The number of GFAP expressing cells decreased by 13.19 ± 2.65 cells/mm^2^, which corresponded to a decrease in the percentage of GFAP-expressing cells of all cells 5.92 ± 1.86% (Fig. 1C). The number of Class III-β-Tubulin expressing cells (neurons) decreased by 10.65 ± 1.41 cells/mm^2^ and the percentage decreased by 4.93 ± 1.31% (Fig. 1D). The decrease in cell numbers could be attributed to a loss in trophic support as reflected by an increase in cell death or a reduction in proliferation. Cell death was quantified using the cell death marker PI and the nuclear stain 4′6-diamidino-2-phenylindole (DAPI) in live cell cultures as shown previously (Zaben et al., 2009). The percentage of non-microglial cells that died (PI^+^IB4^−^), with respect to the total number of non-microglial cells (DAPI^+^IB4^−^), increased from 23.94 ± 2.84% under control conditions to 36.75 ± 3.26% when microglia were removed from culture (Fig. 1E) indicating a trophic effect of microglia in our cell cultures. To address effects on the proliferative population of nestin positive NSPCs, cultures were grown for 3DIV and pulsed with the S-phase marker BrdU for the terminal 8 h before fixation, and the mitotic index was determined. Depletion of microglia resulted in a reduction in the mitotic index of nestin cells from 30.48 ± 1.81% to 20.37 ± 2.35% (Fig. 1F).

### Purified Hippocampal Microglia Are Pro-neurogenic and Increase the Survival and Proliferation of NSPCs

**Figure 2:**
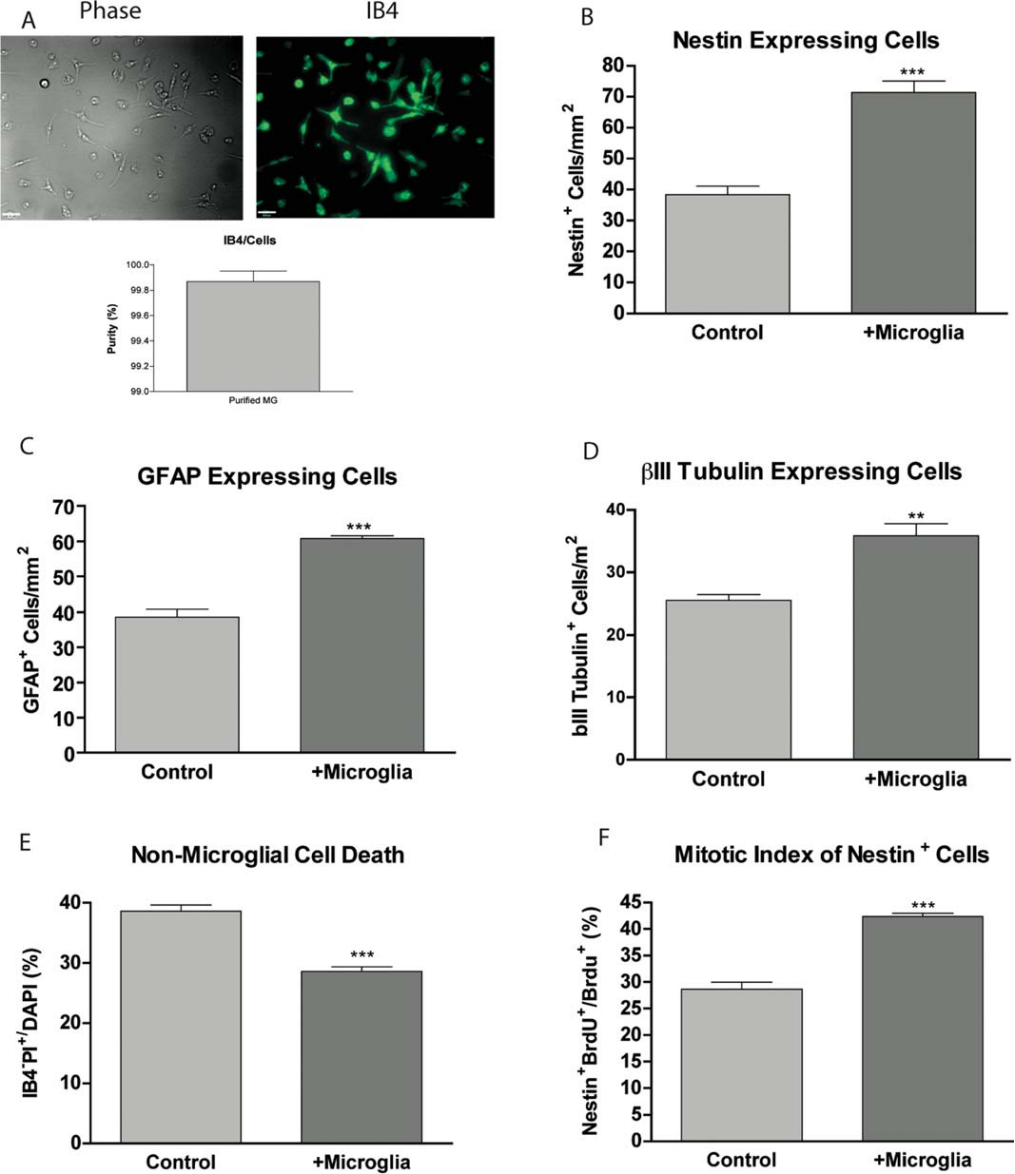
Co-culture of hippocampal progenitor cells with microglia increases their survival as well as the proliferation of nestin-expressing progenitors. A: Isolated microglia are 99.9% pure as determined using IB4 staining (scale bar = 25 μm). B: Purified microglial cell suspension was added to hippocampal cultures at 2 h after plating. Cells were cultured for 5DIV and then fixed and stained for B: nestin, C: GFAP, and D: III-β-Tubulin. E: At 5DIV DAPI, PI, and IB4 were added for 3 h, after which cells were imaged. The proportion of non-microglial cells that are dead (IB4^−^PI^+^/IB4^−^DAPI^+^) was calculated. F: 20 μM BrdU was added to cells at 3DIV for 8 h. Cells were then fixed and stained for BrdU and Nestin. The mitotic index was calculated. **P < 0.01, ***P < 0.001 (Student's *t*-test).

To further examine the effect of microglia, we adapted a protocol from (Giulian and Baker, 1986) to generate 99.9% pure microglial cultures isolated from the postnatal hippocampus (see Materials and Methods). Adding 50,000 cells/cm^2^ of purified hippocampal microglia (Fig. 2A) to hippocampal progenitor cultures resulted in an increase in the number of nestin expressing cells from 38.36 ± 2.78 to 71.40 ± 3.70 cells/mm^2^ (95.50 ± 17.82% increase) (Fig. 2B). The number of GFAP-expressing cells increased from 38.50 ± 2.32 to 60.76 ± 0.74 cells/mm^2^ (59.67 ± 10.25% increase) (Fig. 2C). The number of Class III-β-Tubulin-expressing cells increased from 25.48 ± 0.93 to 35.84 ± 1.92 cells/mm^2^ (40.87 ± 7.24% increase) (Fig. 2D). Consistent with our previous microglial depletion results, the percentage of non-microglial cell death relative to DAPI was reduced from 38.57 ± 1.08% to 28.63 ± 0.74% when microglia were co-cultured with hippocampal progenitors (Fig. 2E). We next studied the effect on the proliferation of nestin-expressing progenitors and found that the mitotic index of nestin-expressing cells increased from 28.64 ± 1.35% under control conditions to 42.37 ± 0.60% when co-cultured with microglia (Fig. 2F).

### Microglia-Conditioned Medium Increases Cell Survival and NPSC Proliferation

**Figure 3:**
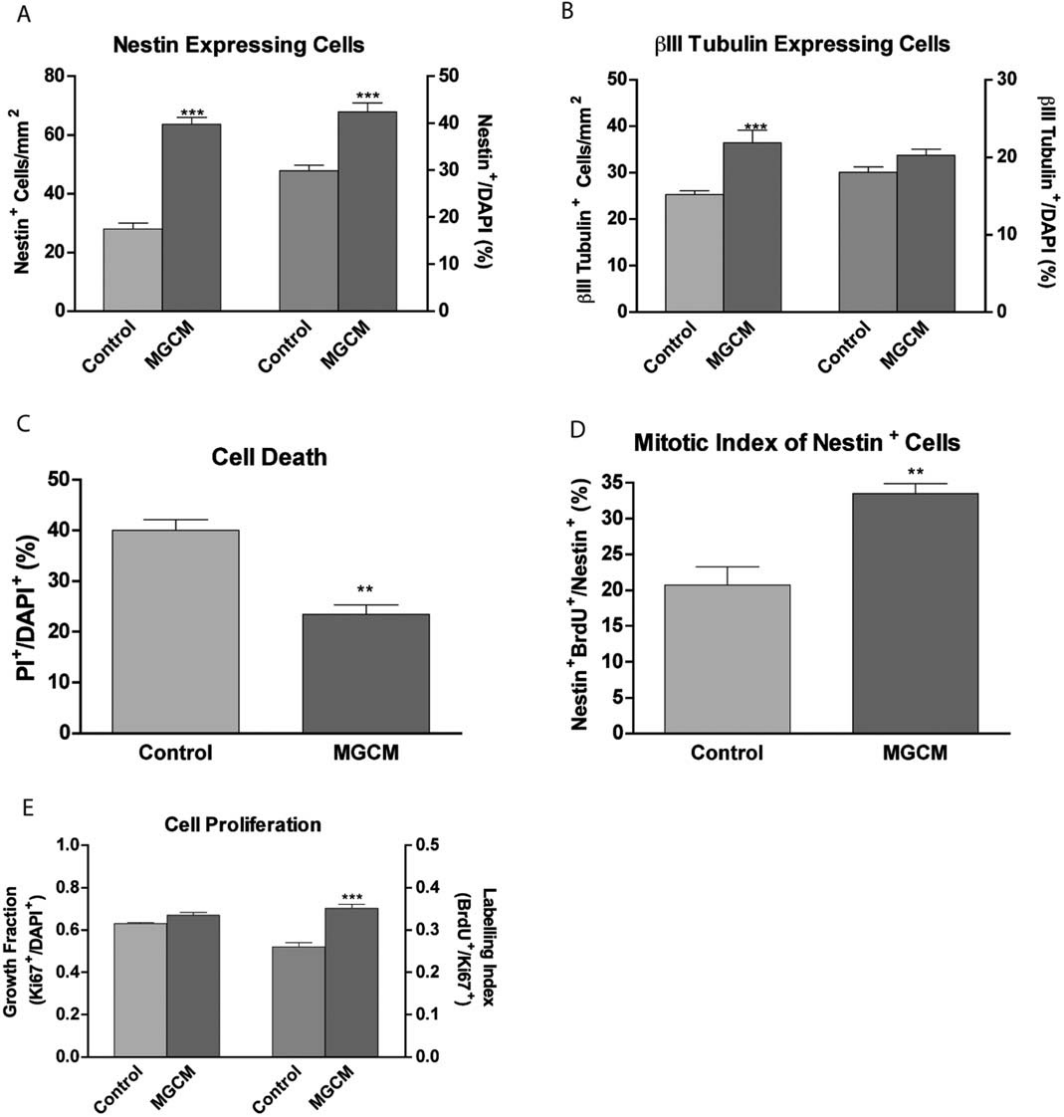
Addition of microglia-conditioned medium to hippocampal progenitor cells increases cell survival and proliferation of nestin-expressing progenitors. Microglia-conditioned medium (MGCM) was added to hippocampal cultures at 2 h after plating. At 5DIV, cells were fixed and stained for A: nestin, B: III-β-Tubulin. Percentages of each cell phenotype with respect to the total cell count (DAPI^+^) were also calculated. C: At 5DIV, DAPI and PI were added for the final 3 h prior to washing and imaging. The percentage of cells that are dead (PI^+^/DAPI^+^) was calculated. D: The mitotic index (expressed as a percentage of nestin^+^ cells that are also BrdU^+^) was calculated by addition of 20 μM BrdU to cells at 3DIV for 8 h. Cells were then fixed and stained for BrdU and Nestin. E: At 3DIV, the growth fraction was calculated based on the percentage of DAPI cells expressing Ki67. The labeling index was calculated by the percentage of Ki67 cells staining for Brdu after an 8 h pulse, **P < 0.01, ***P < 0.001 (Student's *t*-test).

To see if the effects of microglia were due to released soluble factors we applied neat filtered supernatant from the pure microglial cultures to microglia depleted hippocampal cultures from 1DIV to 3DIV. Interestingly, the effects of microglia co-culturing were consistently replicated when filtered microglia conditioned medium was used. The number of nestin-expressing cells increased by 35.65 ± 3.16 cells/mm^2^ and the percentage increased by 12.55 ± 2.25% (Fig. 3A). Although the number of Class III-β-Tubulin expressing cells increased in cultures treated with microglia conditioned medium (by 11.11 ± 2.21 cells/mm^2^), the proportion did not increase significantly (Fig. 3B). As with our co-culture experiments, cell death was significantly reduced (by 16.59 ± 2.80%) in cultures treated with microglia conditioned medium (Fig. 3C). The fact that a proportional increase was seen in nestin-expressing cells while not in Class III-β-Tubulin expressing cells, indicated the possibility that microglia conditioned medium has a proliferative effect on nestin-expressing cells. The proliferative effect on NSPCs was confirmed by the increased mitotic index of nestin-expressing cells from 20.70 ± 2.54% under control conditions to 33.48 ± 1.38% in cultures treated with microglia-conditioned medium (Fig. 3D). We further demonstrated that treatment of cultures with microglia-conditioned medium increased the labeling index (BrdU/Ki67) from 0.26 ± 0.01 to 0.36 ± 0.01 (Fig. 3E). However, the growth fraction (Ki67/DAPI) was unaffected (Fig. 3E), indicating that the proliferative effect was mediated via speeding up the cell-cycle at G1-S-phase transition, and not through recruitment of quiescent cells. The replication of the effects of microglia co-culture by microglia-conditioned medium raised the possibility that microglia exert their effect via released secondary trophic mediators and cytokines, as the filtered supernatant was cell free.

### Pre-treating Microglia with VIP Boosts the Proliferative and Trophic Effects of Microglia on Hippocampal Progenitors

**Figure 4:**
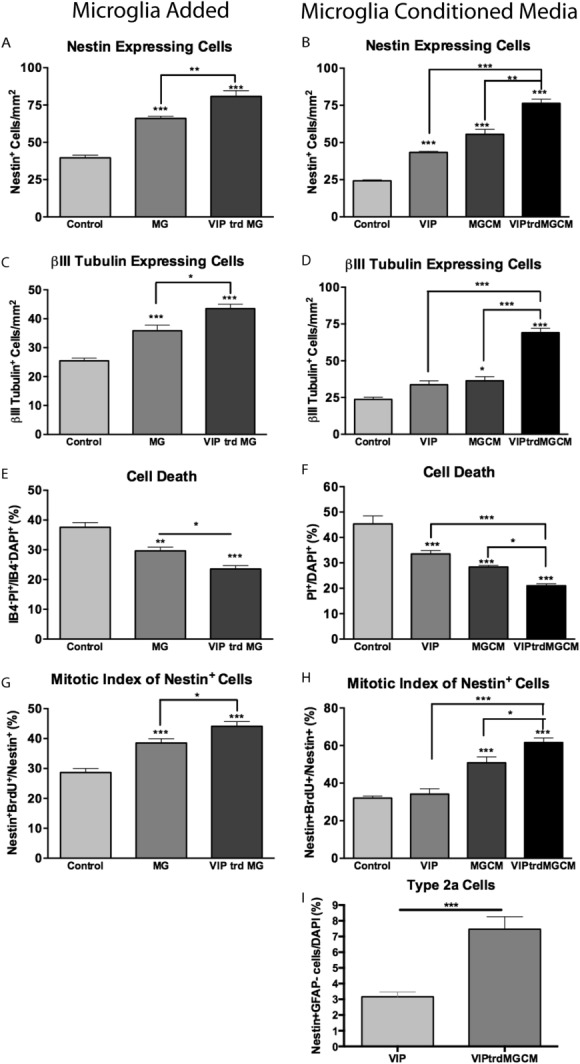
The proliferative and trophic effects of microglia on hippocampal progenitors are significantly enhanced by pre-treating microglia with VIP. Cells were cultured for 5DIV with either standard microglia or VIP (30 nM) treated microglia (24 h exposure) (A, C, E, G). Cells were cultured for 5DIV with either standard MGCM VIPtrdMGCM (B, D, F, H and I). Cells were then fixed and stained for A and B: nestin, C and D: III-β-Tubulin. E and F: At 5DIV IB4, DAPI and PI were added for 3 h prior to one wash and imaging was performed. The proportion of non-microglial cells that are dead (IB4^−^PI^+^/IB4^−^DAPI^+^) was calculated (ANOVA *P* < 0.0001). G and H: 20 μM BrdU was added to cells at 3DIV for 8 h. Cells were then fixed and stained for BrdU and Nestin. The mitotic index (expressed as a percentage of nestin^+^ cells that are also BrdU^+^) was calculated. I: Nestin-positive GFAP-negative cell numbers. *P < 0.05, **P < 0.01, ***P < 0.001 (Student's *t*-test or one-way ANOVA; Bonferroni's multiple comparison test).

To test our hypothesis that VIP modulates the effect of microglia on NSPCs we examined the effect of pre-treating microglia with VIP on NSPCs by adding either VIP pre-treated or vehicle treated microglia or their filtered supernatant to NSPC cultures. Purified hippocampal microglia were cultured for 24 h in fresh medium with 30 nM VIP to generate VIP-treated microglia and filtered supernatant. We found that the number of nestin-expressing cells increased from 66.08 ± 1.50 cells/mm^2^ in cultures with microglia added, to 80.78 ± 3.82 cells/mm^2^ in cultures with VIP-treated microglia added (Fig. 4A). Similarly, cultures treated with MGCM had 55.58 ± 3.32 nestin-expressing cells/mm^2^ while those treated with VIP-treated MGCM had 76.36 ± 2.80 nestin-expressing cells/mm^2^ (Fig. 4B). The number of Class III-β-Tubulin expressing cells increased from to by 40.87 ± 7.24% in cultures treated with microglia and by 71.41 ± 7.52% in cultures with VIP-treated microglia added (Fig. 4C). A similar result was seen with conditioned medium experiments where MGCM increased the number of Class III-β-Tubulin expressing cells by 56.60 ± 19.62% and VIP-treated MGCM increased it by 97.2 ± 9.48% (Fig. 4D). The trophic effect of microglia appeared to be enhanced by pre-treatment with VIP both in co-culture and conditioned medium experiments—cell death reduced by 6.07 ± 1.68% in co-culture experiments (Fig. 4E), and 7.38 ± 1.00% in conditioned medium experiments (Fig. 4F), when microglia were pre-treated with VIP. The proliferation of nestin expressing NSPCs (as indicated by the mitotic index after short pulse BrdU exposure) also increased further in cultures exposed to VIP-treated microglia compared with non-VIP-treated microglia (44.10 ± 1.60% *vs*. 38.54 ± 1.41%) (Fig. 4G). Similar results were found in conditioned medium experiments (Fig. 4H). VIPtrdMGCM significantly increased the number of Nestin-positive GFAP-negative cells (Type 2) from 7.34 ± 0.81 cells/mm^2^ to 22.73 ± 2.4 cells/mm^2^ (Fig. 4I).

### VIP Acts on the VPAC1 Receptor to Boost the Effect of Microglia on NSPC Numbers

**Figure 5:**
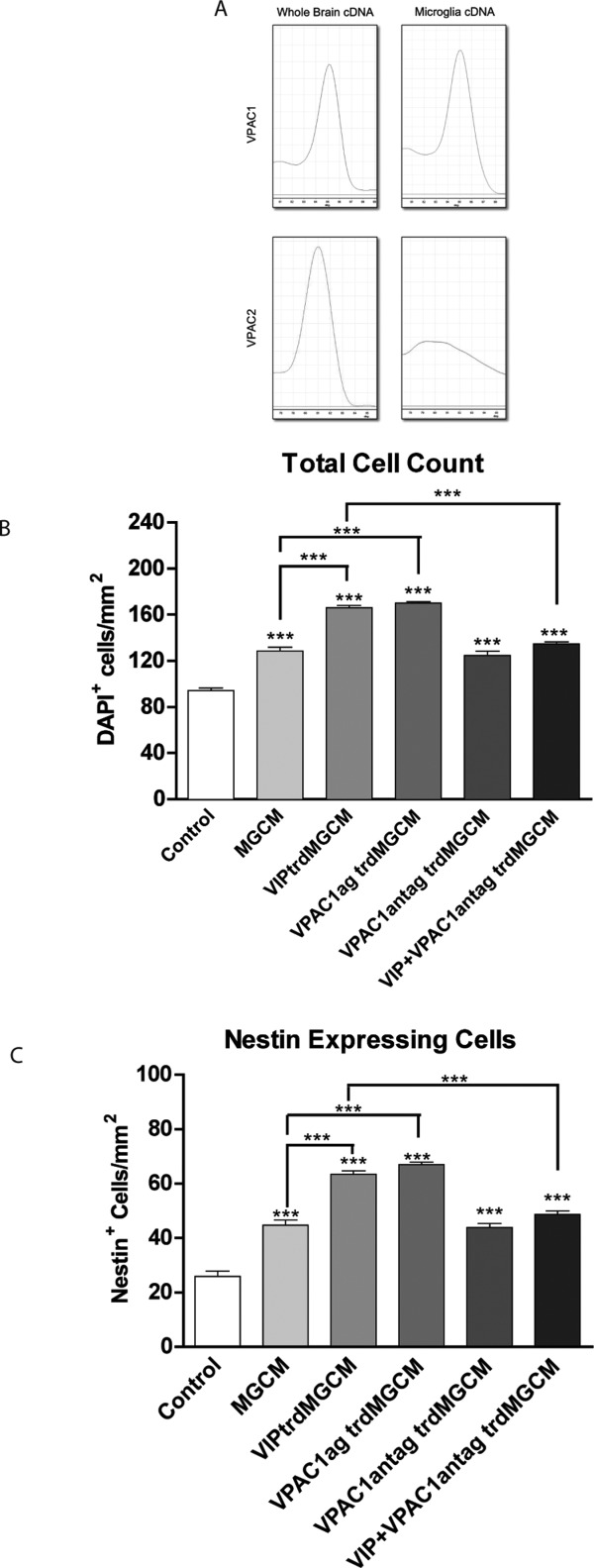
VIP acts on the VPAC1 receptor to increase the trophic effect of microglia on hippocampal progenitors. A: qPCR melt curves of VPAC1 and VPAC2 from cDNA generated from either whole brain RNA or purified hippocampal microglia. For pharmacological studies, hippocampal progenitor cells were cultured for 5DIV under either 1: control conditions, 2: microglia-conditioned medium, 3: VIP (30 nM) -treated microglia-condition medium, 4: VPAC1 (30 nM) agonist-treated microglia-conditioned medium, 5: VPAC1 (1 μM) antagonist-treated microglia condition medium, 6: VIP (30 nM) and VPAC1 antagonist (1 μM) -treated microglia-conditioned medium. Cells were then fixed and stained for B: DAPI, to give total cell counts and C: nestin. *** *P* < 0.001 (one-way ANOVA; Bonferroni's multiple comparison test).

VIP at physiological concentrations (nM) is known to act through the high affinity receptors VPAC1 and VPAC2 receptors (Harmar et al., 1998; Zaben et al., 2009). Using real-time PCR we have shown that VPAC1, but not VPAC2, receptor mRNA is expressed in postnatal hippocampal microglial cultures (Fig. 5A).

To investigate the involvement of VPAC1 in VIP-microglia mediated effect, a selective VPAC1 agonist and selective VPAC1 antagonist were used. Because of its proliferative and trophic effect, addition of MGCM to cultures increased the total cell count (from 94.27 ± 2.04 cells/mm^2^ to 128.4 ± 3.41 cells/mm^2^). This effect was enhanced if microglia were pre-treated with VIP (from 128.4 ± 3.41 cells/mm^2^ to 166.1 ± 2.01 cells/mm^2^). The effect was replicated when microglia were treated with a VPAC1 agonist in place of VIP. Treating microglia with VIP and VPAC1 antagonist appeared to abolish the effect of VIP pre-treatment of microglia, on total cell counts (Fig. 5B).

A similar pattern of results was seen when we looked at the numbers of nestin-expressing cells (Fig. 5C). These data strongly suggest that VIP exerts its effects on microglia via the VPAC1 receptor.

### The VIP/MG Proliferative Effect on Hippocampal Precursors Is IL-4 Mediated

**Figure 6:**
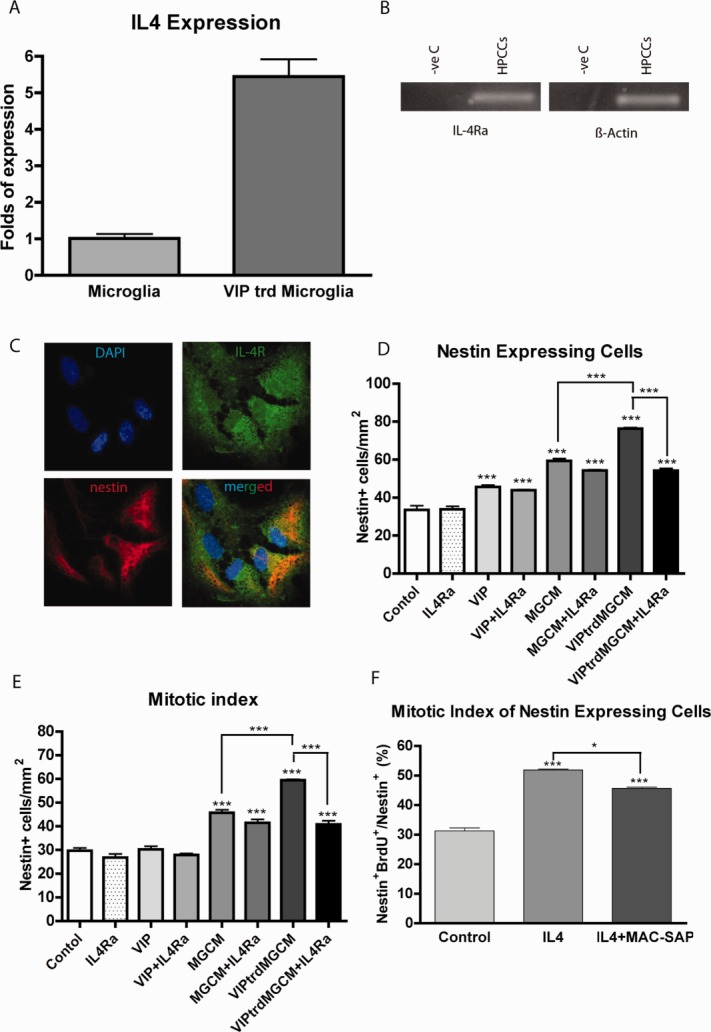
The VIP/MG proliferative effect on hippocampal precursors is IL-4 mediated. A: VIP (30 nM) induces IL-4 gene expression in purified hippocampal microglia cells. B: Hippocampal cells express the IL-4R gene. C: Confocal images showing immunostaining of nestin cells co-expressing IL-4Ra. D and E: 5 DIV exposure to IL-4Ra antibody (10 μg/ml) inhibits the increase in nestin numbers (D) and mitotic index (E) induced by VIPtrdMGCM but not by unstimulated MGCM or direct application of VIP (30 nM) to hippocampal cultures. F: Direct application of recombinant IL-4 (10 ng/ml) for 3DIV onto microglia depleted hippocampal cultures stimulates nestin proliferation even in the absence of endogenous microglia. ***P < 0.001 (one-way ANOVA; Bonferroni's multiple comparison test).

Microglia are known to release soluble factors that affect NSPC survival and proliferation (Cacci et al., 2008; Ekdahl et al., 2009). IL-4 is a key immune cell derived cytokine that can promote learning and memory (Derecki et al., 2010). Using quantitative PCR we found that treating microglia with 30 nM VIP resulted in a fivefold increase in mRNA expression of interleukin-4 (Fig. 6A).

We next sought to identify the presence of the IL4 receptor (IL-4R) in our hippocampal culture system. The expression of IL-4R mRNA was examined in hippocampal cultures at 2 h after plating. The amplification products of our PCR were run on 2% agarose gel stained with ethidium bromide and then visualized with UV light. As shown (Fig. 6B), IL-4R is expressed in our hippocampal cell cultures.

The presence of IL-4R on nestin-positive cells was then examined using immunohistochemistry. Confocal imaging confirmed the expression of IL-4R by nestin-expressing cells at 3DIV (Fig. 6C).

To examine the involvement of IL4 in mediating the proliferative effect of VIP-treated microglia on nestin-expressing cells, hippocampal cultures were treated with microglia-conditioned medium or VIP-treated microglia-conditioned medium in the presence or absence of 10 μg/ml of IL-4Ra, a neutralizing antibody against the alpha subunit of the IL-4 receptor which prevents IL-4 binding, and the mitotic index was determined (no effect of an isotype control antibody was observed, Supp Fig. 1). Blocking IL-4 activity had no significant effect on either the total number of nestin expressing cells (Fig. 6D) nor the mitotic index (Fig. 6E) under control, VIP (30 nM), or microglia conditioned medium conditions. However, compared with VIP-treated MGCM, IL-4 receptor blockade reduced the number of nestin-expressing cells from to 76.25±0.64 to 54.23 ± 1.14 cells/mm^2^. The mitotic index was reduced from 59.51 ± 0.32% in cultures treated with VIP-treated MGCM to 40.83 ± 1.55% in cultures treated with both VIP-treated microglia conditioned medium and the IL-4 receptor antagonist. Thus, the enhanced cell proliferation seen when microglia are pre-treated with VIP appears to be abolished when IL-4 receptors are blocked, suggesting that IL-4 may mediate the proliferative effect of VIP-treated microglia on neural progenitors.

To further determine whether IL-4 has a direct proliferative effect on nestin-expressing progenitors, we treated hippocampal cultures with 10 ng/ml recombinant IL-4 and measured the mitotic index of nestin-expressing cells. The mitotic index increased form 31.27 ± 1.00% under control conditions to 51.88 ± 0.35% under IL-4 treatment. Interestingly, the mitotic index was still raised (45.66 ± 0.40%) when IL-4 was added to cultures treated with MAC-SAP (i.e., depleted of microglia) indicating that IL-4 acts directly on nestin-expressing cells rather than acting indirectly via microglia resident in our cultures (Fig. 6F).

Taken together these findings implicate IL-4 and its receptor system in the modulation of VIP-microglia enhanced hippocampal progenitor cell proliferation in keeping with the hypothesis that IL-4 is an important component in the neuro-immuno-modulation of postnatal hippocampal neurogenesis.

## Discussion

Adult neurogenesis is important for learning and memory, is promoted by neural activity, and is under complex regulation by the immune system, which can be both pro and anti-neurogenic (Deisseroth et al., 2004; Dupret et al., 2007; Ekdahl et al., 2003; Monje et al., 2003; Shors et al., 2001; Snyder et al., 2005; Stone et al., 2011). Microglia, the brains resident immune cell, can either promote or inhibit the proliferation of NSPCs and their differentiation into neurons through the release of cytokines (Aarum et al., 2003; Battista et al., 2006; Butovsky et al., 2006; Cacci et al., 2008; Ekdahl et al., 2003; Monje et al., 2003). Our understanding of the interplay between neural activity, immune function and neurogenesis are limited. Microglia have been shown to express a variety of different receptors for neurotransmitters and neuropeptides (Pocock and Kettenmann, 2007). They have also been postulated to respond to basal and stimulated neuronal activity (Biber et al., 2007). Here, we show that microglia secrete trophic and proliferative factors for NSPCs. Stimulating hippocampal microglia with the peptide neurotransmitter VIP increases production of IL-4 which promotes their trophic and proliferative effects on NSPCs, the latter most likely mediated via IL-4 release. We propose a neuro-immuno-neurogenic pathway where neuronal activity adapts microglial control of neurogenesis by modulating their production and release of cytokines, such as IL-4.

### Microglia Release Soluble Mediators that Promote NSPC Survival and Proliferation

The neurogenic niche is a unique environment that enables ongoing neurogenesis to occur long into adulthood but can be modulated in response to internal and external stimuli. As microglia are found in neurogenic zones they are ideally placed to influence the production of new neurons, but their effects are complex (Ziv et al., 2006). Evidence suggests the type and duration of stimuli are crucial in determining whether their net effect is beneficial or detrimental to neurogenesis (Cacci et al., 2008). Previous studies have approached the role of microglia in neurogenesis but have neglected to use hippocampal microglia, which are distinct in their density and morphology from microglia in other brain regions (Elkabes et al., 1996; Lawson et al., 1990; Phillips et al., 1999). As microglia from different brain regions express different inflammatory profiles (de Haas et al., 2008; Ren et al., 1999), it is realistic to reason that hippocampal microglia which exist in a specialized neurogenic niche may differ in their abilities to regulate neurogenesis compared with microglia derived from other brain regions. In our hippocampal culture system previously used to investigate neurogenesis we found a significant proportion of cells to be microglia (Howell et al., 2005), but we have not previously investigated whether they influence neurogenesis. In these hippocampal cultures, selectively depleting endogenous microglia reduces NSPC proliferation and survival, which results in fewer new neurons. This was reciprocated with an increase in survival, proliferation, and number of immature neurons following addition of purified hippocampal microglia. As in the adult mouse brain 70% of new neurons normally die, our data suggest that microglia have the potential to reduce the amount of immature neuron cell death and thus provide a stimulus for increasing proliferation of the neuroblasts (Biebl et al., 2000; Dayer et al., 2003). Microglia can release both pro and anti-inflammatory cytokines. For example, TGF−β is released following macrophage engulfment of apoptotic cells (Fadok et al., 1998). However, in a co-culture model is not possible to distinguish if the effects seen are via direct cell–cell contact, phagocytosis of inhibitory debris, or via the release of soluble mediators. To address this issue, we added microglia conditioned media directly to NSPC cultures depleted of microglia. Microglia conditioned media enhanced the number and proportion of nestin, cells, and also increased TUJ1 cell number. This suggests that hippocampal microglia release soluble mediators that promote neurogenesis preferentially through stimulating NSPC growth. Although it is now established that microglia can be detrimental or beneficial to neurogenesis depending on how they are activated, these studies have focused on how the adaptive immune system or bacterially derived ligands influences microglia output (Ekdahl et al., 2003; Monje et al., 2003). It has recently been shown that neuronal activity can influence immune function and vice versa (McAllister and van de Water, 2009). Therefore, we investigated how neuropeptides, commonly found in dentate gyrus inter-neurons innervating the neurogenic niche, can modulate microglia's neurogenic benefits.

### VIP Augments Microglial Trophic and Proliferative Effects via VPAC1 Receptors

VIP is a hippocampal neuropeptide localized in the CA1, CA3, and dentate gyrus of the hippocampus (Loren et al., 1979). Our group and others have demonstrated that VIP can directly affect NSPC maturation (Scharf et al., 2008; Zaben et al., 2009). It is also one of the best-studied immunomodulaotry neuropeptides known to down regulate pro-inflammatory cytokine release from LPS stimulated microglia and enhance anti-inflammatory cytokine release (Ganea et al., 2006; Leceta et al., 2000). Here, we show that in addition to VIP's direct effect on nestin positive NSPCs (Zaben et al., 2009), simulating hippocampal microglia with physiological concentrations of VIP boosts microglia's pro-neurogenic properties. VIP stimulates microglia to release soluble mediators which enhance NSPC proliferation (Fig. 4H) and cell survival in hippocampal cultures (Fig. 4F). We did not determine whether this trophic effect was on all cell types or was specific to proliferating cells, but focussed instead on the proliferative effect here. At physiological concentrations, VIP can act through either VPAC1 or VPAC2 receptors. Consistent with previous studies on macrophages and microglia our purified hippocampal microglia express VPAC1 but not VPAC2 genes (Delgado et al., 2002). Furthermore our pharmacological data support VIP's action to be through VPAC1. This suggests during periods of high hippocampal activity such as learning, the release of VIP from interneurons can have a dual effect on neurogenesis by directly promoting nestin positive NSPC expansion via VPAC2 receptors and indirectly via boosting the release of proliferative factors from microglia through VPAC1 receptors.

### VIP-Treated Microglia's Pro-neurogenic Phenotype Is Due to Increased IL-4 Expression and Release

Microglia can dynamically adopt different expression profiles of cytokine and inflammatory mediators depending on the length and type of stimuli. Although the exact profile is also dynamic, a consensus is building toward a detrimental pro-inflammatory and beneficial anti-inflammatory phenotype. VIP can modify the inflammatory response of macrophages and microglia stimulated with LPS (Delgado et al., [Bibr b16],[Bibr b15]). Therefore, we sought to determine the molecular basis of VIP's pro-neurogenic action. In hippocampal microglia cultures, VIP up-regulates IL-4 gene expression. IL-4 derived from the adaptive immune system is important for learning and memory by preventing development of a pro-inflammatory phenotype (Derecki et al., 2010). In addition, microglia expressing IL-4 are neuroprotective (Park et al., 2005). Using a neutralizing antibody against the alpha component of the IL-4R which blocks IL-4 binding, we show that it had no effect on unstimulated microglia conditioned media but blocked the additional enhancing effect of VIP-treated microglia conditioned media. In addition, VIP also down regulated microglia TNF−α gene expression (not shown), a pro-inflammatory cytokine known to inhibit neurogenesis (Liu et al., 2005). Therefore, VIP simultaneously can up-regulate pro-neurogenic factors while down regulating anti-neurogenic components. This data suggests that VIP, which is released by GABAergic interneurons under specific firing conditions, could influence the brains endogenous immune profile to promote neurogenesis. Recent studies have confirmed that GABAergic hilar interneurons modulate neurogenesis via the release of GABA which both promotes the maturation of newly born neurons (Markwardt et al., 2011) and maintains the population of radial glia-like stem cells in a quiescent state (Song et al., 2012). Although the latter study showed no effect of VIP releasing interneurons on radial glia-like cells, this is entirely consistent with our findings, as the VIP treatment of microglia had its predominant effect on nestin positive but GFAP-negative cells (Fig. 4I) consistent with an effect on Type 2a (transient amplifying cells) NSPCs (Steiner et al., 2006) which are the progeny of both themselves and of radial glia-like NSPCs. This Type 2a cell population is also the target of the direct effects of VIP (Zaben et al., 2009) which are non-proliferative and mediated via the VPAC2 receptor. Thus, VIP acting directly on Type 2a cells maintains their population pool by promoting symmetrical cell division and survival without altering their proliferation rate. VIP also acts indirectly via a VPAC1 receptor mediated effect on microglia promoting increased IL-4 production and therefore increased Type 2a NSPC proliferation to increase neurogenesis. We have shown that these direct and indirect effects are distinct, as VIP alone had no effect on the proliferation of nestin-positive cells (Figs. 4H and 6E) and while IL-4R blockade did not alter the VIP effect on nestin cells or their proliferation, it did reduce the VIP enhancement of MGCM to that of untreated MGCM (Fig. 6D,E). Furthermore, a significant effect of VIP on nestin cells remained after depleting the cultures of endogenous microglia (data not shown).

We found evidence of a proliferative effect of IL-4 that is not dependant on the presence of endogenous microglia (Fig. 6F). This may be a direct effect given our immunohistochemical detection of IL-4R expression on nestin-positive cells in culture. Although the addition of IL-4R blocking antibody abolished the VIP/MG-mediated effect, it did not abolish the effect of unstimulated MGCM. The enhancement of neurogenesis by IL-4 stimulated MG has been reported to be partly mediated via IGF (Butovsky et al., 2006) and BDNF (Derecki et al., 2010) and IL-4 may therefore have complex effects. Interestingly, IL-4 released from meningeal T Cells has beneficial effects on learning and memory on Morris Water Maze testing and IL-4−/− mice exhibit severe learning deficits [for review see Gadani et al. (2012)]. VPAC1 receptor modulation of microglial phenotype to support hippocampal neurogenesis may therefore be of therapeutic relevance to conditions combining cognitive decline and neuro-inflammation such as temporal lobe epilepsy, Alzheimer's, and other neurodegenerative diseases.

## Conclusion

This study demonstrates a novel neuroimmune-neurogenic pathway that may be important for promoting hippocampal neurogenesis. We provide the first evidence that VIP, a local circuit neuropeptide transmitter found in the dentate gyrus, enhances the ability of microglia to increase NSPC survival and proliferation via an IL-4 mediated mechanism.
