# Supplementary figures and images for: Microglial VPAC1R mediates a novel mechanism of neuroimmune-modulation of hippocampal precursor cells via IL-4 release
Source: Glia. 2014 Jun 18;62(8):1313–27. doi: 10.1002/glia.22682 (PMC4336555; doi:10.1002/glia.22682)

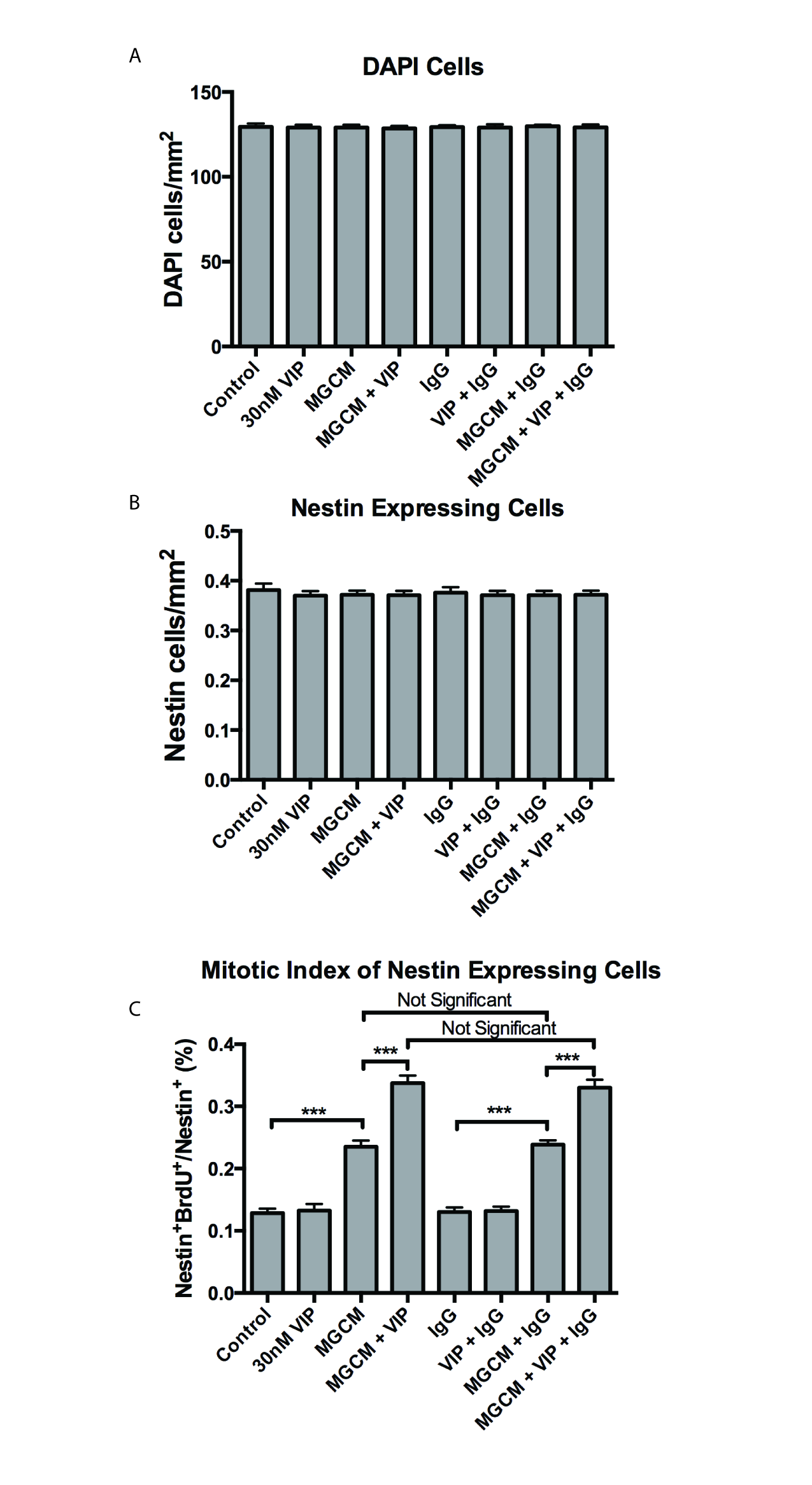

Supplement: Supplementary file 1 — Supplementary Information [file glia0062-1313-sd1.tif]
